# The Arduous Path to Drug Approval for the Management of Prader–Willi Syndrome: A Historical Perspective and Call to Action

**DOI:** 10.3390/ijms241411574

**Published:** 2023-07-18

**Authors:** Deepan Singh, Jennifer L. Miller, Edward Robert Wassman, Merlin G. Butler, Allison Foley Shenk, Monica Converse, Maria Picone

**Affiliations:** 1 Department of Psychiatry, Maimonides Medical Center, Brooklyn, New York, NY 11219, USA; 2 Department of Pediatrics, Division of Pediatric Endocrinology, University of Florida, Gainesville, FL 32611, USA; millejl@peds.ufl.edu; 3 TREND Community, Philadelphia, PA 19103, USA; drbobwassman@gmail.com (E.R.W.); alifoleyshenk.trend@gmail.com (A.F.S.); maria@trend.community (M.P.); 4 Departments of Psychiatry & Behavioral Sciences and Pediatrics, University of Kansas Medical Center, Kansas City, KS 66160, USA; mbutler4@kumc.edu

Prader–Willi syndrome (PWS) is a neuroendocrine genetic disorder resulting from the loss of paternally expressed imprinted genes in chromosome 15q11-q13 [1,2]. Although characterized most prominently by life-threatening hyperphagia and obesity, PWS can also be accompanied by a multitude of other issues, including growth and other hormone deficiencies, behavioral problems, skin picking, abnormal body composition [3,4], and sleep disruption [5,6]. Despite the well-established burden of illness and the social costs of PWS, the only medication that has been approved by the Food and Drug Administration (FDA) for the management of this disorder is recombinant human growth hormone (rhGH), which was approved in 2000 [7,8]. However, since then, every PWS clinical trial that has reached phase 3 has failed to achieve final approval. Here, we discuss some insights into the challenges associated with the approval of drugs to treat PWS.

The increased awareness of PWS has led to earlier diagnosis and resultant intervention and treatment [9]. With the approval and introduction of rhGH during early development and the implementation of individualized dietary and exercise plans [10], progress has been made in improving stature and body composition associated with PWS; however, limited progress has been made regarding neurobehavioral manifestations of the disease. Although PWS was once considered a disorder marked by cognitive impairment, some individuals with PWS now attend mainstream school classrooms and have a wealth of options for attending college, demonstrating the potential for more independent living for those with this disorder.

Despite these incremental advances, hyperphagia, its repercussions, and behavioral dysregulation in PWS remain severe and life-threatening. Hence, the importance of novel drug development and approval cannot be overstated. A recent review by Mahmoud et al. [11] detailed clinical trials of multiple proposed therapeutics for PWS and highlighted other potential prospects for evaluation. Thus far, these therapies and others have mostly been discontinued or failed to receive approval from the FDA.

The challenges inherent in rare disease clinical trials are well known. Notably, the intrinsic limitation in sample size is exponentially compounded by the geographical scale needed for recruitment and enrollment for studies to attain sufficient power to reach statistical significance.

To address these challenges, the FDA has used its regulatory flexibility to its advantage, ultimately approving drugs to treat many illnesses, predominantly hematological–oncological diseases and, more recently, neurodegenerative disorders such as Alzheimer’s disease and amyotrophic lateral sclerosis (ALS). Such regulatory discretion applies when a disease is serious, life-threatening, and has few or no therapies available. All of these factors apply to PWS, and any therapeutic approval pathway for this syndrome should therefore be eligible for “flexibility”. This would allow for the acceptance of greater-than-usual uncertainty about the effi-cacy of a treatment modality for an unmet medical need. However, the FDA has yet to apply its regulatory flexibility regarding medications developed for this population.

Arguments posed by the FDA include a lack of sufficiently well-described mechanisms of action for agents and inadequate direct or surrogate study endpoints. The lack of established biomarkers in PWS that serve as potentiating surrogate endpoints remains a barrier to successful studies, which historically have been heavily influenced by subjective caregiver questionnaires. The result is an inherent rigidity to the regulatory flexibility process and no path forward to address the unmet needs of PWS.

Under the current regulatory construct, in which reliance on biomarkers and the requirement of prestated narrow goals cannot be changed to include other relevant clinical improvements, the approval of new treatments for PWS remains scarce. We contend that recent clinical trials that failed to meet primary endpoints still had significant off-target benefits for participants, often with minimal to no adverse effects. A recent example is carbetocin and its effects on PWS-related anxiousness and hyperphagia [12]. Additionally, diazoxide choline controlled-release (DCCR) recently demonstrated significant improvements in hyperphagia, as well as in anxiety, depression, and compulsive and self-injurious behavior; however, the top-line analysis was negatively affected by the COVID-19 pandemic, which caused the overall analysis to miss its primary endpoint. Long-term data continue to show improvements in hyperphagia as well as several behavioral parameters, and comparisons with a contemporaneous natural history study have shown the significant benefits of DCCR treatment [13]. The fact that the significant beneficial effects of this agent are disastrously overshadowed by the rigid analytical process in this setting is a major concern of the patient–caregiver–clinical community.

Many manifestations of PWS have multidimensional and complex etiologies, including genetic factors that remain poorly understood or characterized. Some therapeutic agents or approaches may focus on physical findings or comorbidities such as obesity and abnormal body composition, but not on behavioral manifestations, which also impair quality of life across many domains [3]. The complexity of PWS and history of failed trials are leading to the further marginalization of patients with PWS and risk causing a feeling of disinterest among the scientific and pharmaceutical communities; the barriers to treatment might seem insurmountable and not worth pursuing.

The future envisioned by the patient–caregiver–clinician community is one in which people with PWS can make meaningful contributions to society and have purposeful lives of their own without having to face as-yet-unaddressed biological and psychological challenges. This is within reach in the near future if the scientific, clinical, industry, and regulatory communities work toward this common goal. For this to happen, we must remove obstacles to the approval of reasonable and effective treatments for people with PWS. We strongly recommend that the FDA apply the broadest regulatory flexibility to the statutory standards and use clinically grounded scientific judgment, as we discussed, paired with an understanding that patients and their families are willing to accept greater risk and uncertainty for the opportunity to ameliorate the severely disabling, life-threatening symptoms of PWS.
